# Editorial: Post COVID-19: analysing and addressing the challenges faced by patients following intensive care treatment for COVID-19

**DOI:** 10.3389/fsoc.2023.1332156

**Published:** 2023-11-28

**Authors:** Vincenzo Auriemma, Gennaro Iorio, Giuliana Scarpati, Ornella Piazza

**Affiliations:** ^1^Department of Political and Social Study, University of Salerno, Fisciano, Italy; ^2^Department of Medicine, Surgery and Dentistry, University of Salerno, Baronissi, Italy

**Keywords:** doctor–patient, intensive care, post COVID-19, social wellbeing, psychic wellbeing

## Introduction

This Research Topic has aimed to reflect the significant consequences of the pandemic for COVID-19 survivors; therefore, it offers readers the opportunity to understand all those factors that have influenced and continue to influence patients' distress and opportunities for recovery, with the aim of best ensuring that the consequences of the pandemic do not develop into chronic injuries.

The key concept throughout the Research Topic was transdisciplinarity, as reflected both in the scientific differentiation of the papers received and in the very organization of the various authors within each paper. So, given the nature of the topic, which falls between medicine, sociology, social work and psychology, it was possible to obtain a more comprehensive assessment of the complexity of the current situation (Auriemma et al., 2023).

Although the health perspective focuses primarily on understanding the physiological aspects of COVID-19, it was possible to capture, through the papers presented, the different ways in which patients were able to restore their wellbeing not only physiologically, but also socially, especially through the support they received from medical professionals as many papers describe and from local health services after discharge from intensive care units (ICUs).

## Review and opinion contributions

Among the various works that confirmed and lent luster to our main objective, we certainly find Di Rosa's review, whose aim was to reflect on the specific area of the interaction between health care and social care (and vice versa). This field, as obvious as it is, does not always receive the right attention, whose roles increasingly go by the wayside despite the enormous amount of work being done. Therefore, the author showed how medicalization in emergency management has undermined or, at least, weakened the comprehensive approach to the person and vulnerability profiles that should inspire social and health integration (Di Rosa). The author, also described the relationship that exists between health systems and social systems and the effects of the COVID-19 pandemic on it, pointing out and specifying how much that weak link that, especially in certain parts of the world, was present has been undermined.

By reading Deng's text, however, it is possible to delve, in small steps, into the other focus of our call, that of physiological care processes. Indeed, it is possible to understand how the early application of prone ventilation, for COVID-19 patients, offered a survival advantage, all of which generated, as consecon a lower expected mortality in patients with severe ARDS (Deng and Zou).

## Empirical results: quantitative data and original research

The empirical work section opens with a very interesting paper by Agnoletti et al. who present readers with an innovative and functional method for coping with the increase in intensive care unit beds related to pandemic COVID-19, demonstrating the feasibility and efficiency of a dynamic model of hospital reorganization.

The aim of the study by Wang et al. was to explore the application and effect of the “WeChat cloud service” in the emergency intensive care unit. A kind of tele-medicine, or, at least, tele-support. The research was conducted on 774 patients admitted within the intensive care unit between February 2020 and June 2021. The authors pointed out that, there was a significantly better situation due to lower costs and lower delirium situations. Claiming that the “WeChat cloud service” was helpful in preventing and controlling coronavirus disease 2019 during the outbreak and improving patient experience (Wang et al.).

Instead, authors Snoubar et al., proposed an article starting with a description of the qualitative-quantitative research conducted on the effect of COVID-19 fear toward the future. They analyzed 204 Turkish social workers who were engaged in the front lines against the pandemic. In general, social workers were found to be extremely concerned about contracting COVID-19. However, the authors also pointed out that female social workers had a greater fear of contracting the infection than males. Social workers and frontline committed health workers can use these findings to develop effective intervention programs reduce fears related to COVID-19 (Snoubar et al.).

To enrich our review, it is also possible to read the work of Zulbaran-Rojas et al. The authors present work that investigates the consequences of being in the intensive care unit for long periods of time. Dwelling on one risk in particular, namely the deconditioning of lower extremity muscles, especially in critically ill patients. The study is described as a double-blind, randomized controlled trial through which the safety and efficacy of electrical stimulation in the lower limbs was examined. Therefore, the researchers' goal was to have empirical evidence in the use of electrostimulation to prevent muscle decay (Zulbaran-Rojas et al.).

Another very interesting study for our call is the work of Naorungroj et al. The authors described the characteristics and outcomes of intrahospital mortality of patients hospitalized for COVID-19. This paper is presented as a retrospective review on the medical records of patients with COVID-19 infection admitted to the intensive care unit of Siriraj Hospital between January 2020 and December 2021. The authors hypothesized a strategy based on appropriate selection of patients to be admitted to the ICU and to implement solutions to limit disease progression to prevent intubation (Naorungroj et al.).

One of the most outstanding studies in this call is the descriptive study by Yoo et al. The authors conducted interviews with caregivers of patients admitted to intensive care units during the COVID-19 pandemic. Their goal was to analyze the impact of listening to music on their psychological wellbeing. To collect this information, three questionnaires were administered, the first being the Korean version of the Center for Epidemiologic Studies Depression Scale, and the second being the World Health Organization Quality of Life Scale. Finally, a third, *ad-hoc* constructed questionnaire was used with the aim of collecting information on participants' engagement in musical activities, thus generating a data set that led to interesting results (Yoo et al.).

Regarding the cross-sectional study by Habibi Asgarabad et al. we note how the aim was to assess the validity and reliability of the General Health Questionnaire, characterized by 12 items and administered to patients hospitalized with COVID-19 in 2020. The authors pointed out that among the factorial models, using the three-factor model (successful coping, self-esteem and stress) proved to be the most suitable. So, overall, the results revealed some very interesting data, which had only been hypothesized before. That is, mental distress in patients with COVID-19 is related to high perceived stress and, more importantly, low sleep quality, which is easy to hypothesize because of the noise and intensive care units, but interesting to highlight with empirical data (Habibi Asgarabad et al.).

Within our call we find, also, an experimental study by Maslova et al., which was conducted in the post-COVID-19 paradigm to assess the quality of life after 9 months after leaving the ICU of critically ill patients. Two hospitalization conditions were analyzed, the first involving the use of medical oxygen for therapy and the second involving the non-use of medical oxygen for therapy in addition to outpatient treatment. This represents one of the first studies in the current literature to report the quality of life of patients who responded to treatment 9 months after COVID-19 (Maslova et al.).

Another very interesting study is represented within the work of Kuryllo et al. The authors' goal was to observe how patients admitted to the intensive care unit may exhibit muscle weakness up to a year or more after discharge. Within this study was analyzing neuromuscular progression by distinguishing the results between women and men, however, the study found no sex differences in the parameters assessed in the 3- to 6-month follow-up; the significant difference, however, was found in the 6- to 12-month follow-up (Kuryllo et al.).

The study by Hajkova et al. examined the impact of anxiety and depression symptoms during the first phase of pandemic COVID-19. Therefore, the authors highlighted behavioral, cognitive and emotional changes in the Czech population. The authors' goal was, therefore, to show what has been widely hypothesized, namely, that increased anxiety and depression are symptoms that have characterized the experience of many due to loneliness and reduced close relationships (Hajkova et al.).

The study protocol by Sum et al. observes the residual symptoms manifested by patients in the post-acute and rehabilitation stages include fatigue, dyspnea and insomnia. The double-blind, randomized, placebo-controlled study aimed to evaluate the efficacy and safety of the combination of the two formulas [named “COVID-19 Rehab Formula (CRF)”] in treating the residual symptoms of COVID-19 (long COVID). In addition, evaluating the efficacy and safety of CRF in treating residual symptoms of COVID-19 with a scientifically rigorous design (Sum et al.).

The clinical study by Chi et al. explored the risk factors associated with postoperative hypoxemia in elderly patients recovered from COVID-19 disease and undergoing surgery for hip fracture in the short term. The authors conducted the study within three hospitals in China and found statistically significant differences among patients, and also followed the classification of the American Society of Anesthesiologists by comparing it with the presence of sputum symptoms, preoperative hypoxemia, and pulmonary inflammation from chronic obstructive pulmonary disease. So, it is interesting to note empirically how secondary risks can seriously affect respiratory disease (Chi et al.).

## Conclusion

The main objective of this call, which we felt was fully achieved, was to highlight some of the insights that can be gained from a transdisciplinary exploration in the analysis of patients and health care and non-health care personnel during COVID-19. This allowed, in this way, to generate a pool of research from around the world, highlighting the different ways of operating, assessing and operationalizing the same disease. Consequently, it is important to analyze and delve into any type of theory while avoiding dwelling only on those theories that reflect on understanding others as only a matter of biological input, as the aspect of cultural interaction remains at the core of any discourse. We live in a historical period where isolated sectorization does not lead to any interesting discoveries, so the sciences need transdisciplinarity as m, as seen in this Research Topic, contributing to a common knowledge that can place the person at the center of all scientific discourse, avoiding scientistic reductionism and allowing researchers and scholars to draw on the broadest possible sources. In conclusion, the wonderful and ambitious goal that we editors from distant disciplines had set for ourselves and which the authors of each paper masterfully fulfilled, we must emphasize that today, despite the post-pandemic, it is possible to reflect from this to face the new challenges that the future holds. Mixing the techniques used in these papers, rather than emphasizing a different one than has always been used, to compare the results. So, transdisciplinarity as a deeper way of approaching science and future research.

## Author contributions

VA: Writing—original draft, Writing—review & editing. GI: Writing—original draft, Writing—review & editing. GS: Writing—original draft, Writing—review & editing. OP: Writing—original draft, Writing—review & editing.
